# Latrophilins as Downstream Effectors of Androgen Receptors including a Splice Variant, AR-V7, Induce Prostate Cancer Progression

**DOI:** 10.3390/ijms25137289

**Published:** 2024-07-02

**Authors:** Yuki Teramoto, Mohammad Amin Elahi Najafi, Takuo Matsukawa, Adhya Sharma, Takuro Goto, Hiroshi Miyamoto

**Affiliations:** 1 Department of Pathology & Laboratory Medicine, University of Rochester Medical Center, Rochester, NY 14642, USA; tera1980@kuhp.kyoto-u.ac.jp (Y.T.); mohammadamin_elahinajafi@urmc.rochester.edu (M.A.E.N.); ashar49@u.rochester.edu (A.S.); tak-uro@uro.med.tohoku.ac.jp (T.G.); 2 James P. Wilmot Cancer Institute, University of Rochester Medical Center, Rochester, NY 14642, USA; 3 Department of Urology, University of Rochester Medical Center, Rochester, NY 14642, USA

**Keywords:** ADGRL, androgen receptor, AR-V7, latrophilin, latrotoxin, prostate cancer

## Abstract

Latrophilins (LPHNs), a group of the G-protein–coupled receptor to which a spider venom latrotoxin (LTX) is known to bind, remain largely uncharacterized in neoplastic diseases. In the present study, we aimed to determine the role of LPHNs in the progression of prostate cancer. We assessed the actions of LPHNs, including LPHN1, LPHN2, and LPHN3, in human prostate cancer lines via their ligand (e.g., α-LTX, FLRT3) treatment or shRNA infection, as well as in surgical specimens. In androgen receptor (AR)-positive LNCaP/C4-2/22Rv1 cells, dihydrotestosterone considerably increased the expression levels of LPHNs, while chromatin immunoprecipitation assay revealed the binding of endogenous ARs, including AR-V7, to the promoter region of each LPHN. Treatment with α-LTX or FLRT3 resulted in induction in the cell viability and migration of both AR-positive and AR-negative lines. α-LTX and FLRT3 also enhanced the expression of Bcl-2 and phosphorylated forms of JAK2 and STAT3. Meanwhile, the knockdown of each LPHN showed opposite effects on all of those mediated by ligand treatment. Immunohistochemistry in radical prostatectomy specimens further showed the significantly elevated expression of each LPHN in prostate cancer, compared with adjacent normal-appearing prostate, which was associated with a significantly higher risk of postoperative biochemical recurrence in both univariate and multivariable settings. These findings indicate that LPHNs function as downstream effectors of ARs and promote the growth of androgen-sensitive, castration-resistant, or even AR-negative prostate cancer.

## 1. Introduction

Prostate cancer has represented one of the most common malignancies, and the number of worldwide cancer-related deaths appears to be considerably increasing (e.g., 307,500 in 2012 [1], 375,304 in 2020 [2]). Although definitive therapy, such as radical prostatectomy, can offer a cure in most patients with localized disease, they have a considerable risk of developing recurrent disease for which adjuvant therapy is required [3,4]. More critically, those with advanced hormone-naïve prostate cancer who are usually sensitive to androgen deprivation therapy eventually develop castration-resistant disease for which treatment options are currently limited [5,6]. Meanwhile, the activity of androgen receptor (AR), a member of the nuclear receptor superfamily, is well known to be associated with the outgrowth of even castration-resistant prostate cancer. In particular, splice variants of AR lacking its ligand-binding domain, such as AR-V7, have been extensively studied as an underlying mechanism of castration resistance [5,6,7]. Accordingly, downstream effectors of AR signaling are potential therapeutic targets for hormone-dependent and/or castration-resistant diseases.

Latrophilins (LPHNs), initially isolated as protein(s) to which a neurotoxin as a component of the venom from widow spiders (e.g., *Latrodectus genus*), latrotoxin (LTX), binds [8,9], are a group of the G-protein–coupled receptors consisting of three members, LPHN1, LPHN2, and LPHN3 [10]. In humans, LPHN1 and LPHN3 have been found to be enriched in the brain, while LPHN2 appears to be more ubiquitously expressed [10,11,12]. Although the biological functions of LPHNs are far from being fully understood, alterations in their encoded genes (i.e., *ADGRLs*), particularly *ADGRL3*, have been linked to susceptibility to attention deficit hyperactivity disorder [10,13]. Interestingly, attention deficit hyperactivity disorder is diagnosed approximately three times more often in boys than in girls, and prenatal exposure to excess androgens has been shown to increase its risk [14,15], implying an association of LPHNs with AR signaling. Nonetheless, the involvement of LPHNs in neoplastic diseases remains largely untested. The present study aimed to determine whether LPHNs could serve as downstream effectors of AR and could thereby promote the growth of prostate cancer.

## 2. Results

### 2.1. Effects of Androgen on the Expression of LPHNs

**Figure 1:**
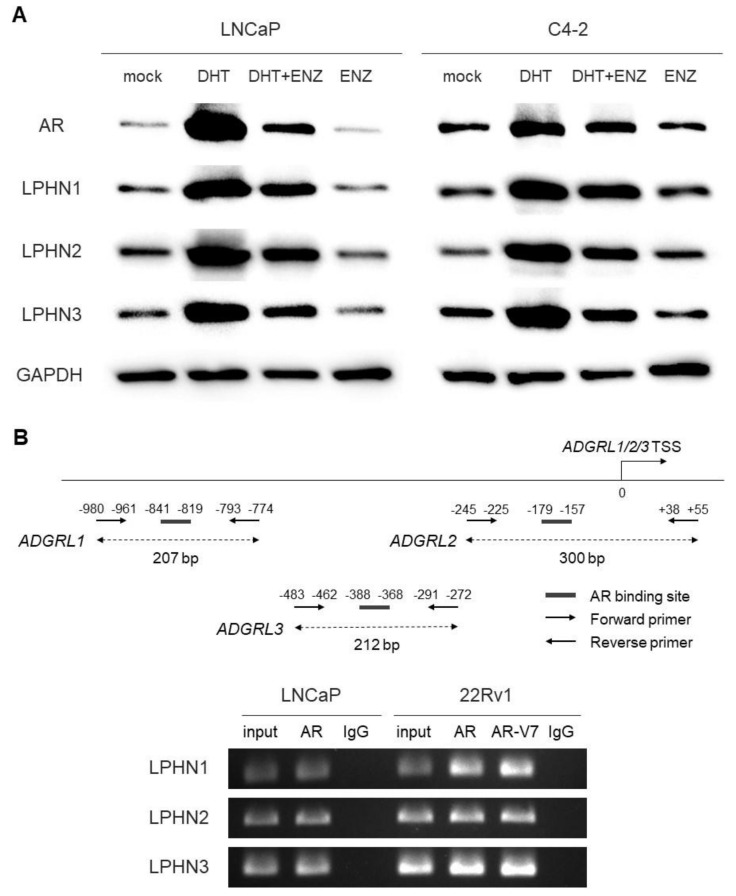
Effects of androgen/AR on LPHNs in prostate cancer cells. (**A**) Western blotting of AR (full-length), LPHN1, LPHN2, and LPHN3 in LNCaP and C4-2 cultured for 48 h with ethanol (mock), 10 nM DHT, and/or 1 µM ENZ, as indicated. GAPDH served as a loading control. (**B**) The ChIP assay, using LNCaP/22Rv1 cell lysates immunoprecipitated with an anti-AR (full-length or AR-V7) or IgG (as a negative control). The DNA fragments were PCR amplified with a set of primers specific to the promoter of *ADGRL1*, *ADGRL2*, or *ADGRL3*, and the PCR products were electrophoresed on 1% agarose gel. Fractions of the mixture of a protein–DNA complex (i.e., 1% of total cross-linked, reserved chromatin prior to immunoprecipitation) were used as “input” DNAs.

We first examined the expression of LPHNs in five human prostate cancer lines. Western blot detected LPHN1/LPHN2/LPHN3 signals in all the cell lines, and the levels were higher in AR-positive cells (i.e., LNCaP, C4-2, 22Rv1) than in AR-negative cells (i.e., DU145, PC-3) (Supplementary Figure S1). We then assessed the effects of androgen [i.e., dihydrotestosterone (DHT)] and anti-androgen [i.e., enzalutamide (ENZ)] on the expression of LPHNs in LNCaP and C4-2. DHT treatment (vs. mock treatment) considerably induced the levels of LPHN1/LPHN2/LPHN3 expression in both lines, which were at least partially restored by ENZ treatment (Figure 1A).

A bioinformatics-driven search identified putative AR binding sites in the promoter regions of *ADGRL1*, *ADGRL2*, and *ADGRL3*. We therefore investigated whether full-length AR and/or AR-V7 could regulate the expression of LPHNs, using a chromatin immunoprecipitation (ChIP) assay (Figure 1B). DNA fragments from LNCaP or 22Rv1 cells immunoprecipitated with an anti-AR antibody or normal IgG were amplified by PCR with sets of primers specific for *ADGRL* promoters. The PCR products for all potential binding sites could be visualized from those precipitated by the AR antibody, but not control precipitants, indicating the interactions of full-length AR or AR-V7 with the *ADGRL1*/*ADGRL2*/*ADGRL3* promoter.

### 2.2. Effects of LPHN Ligand Treatment or Knockdown on Cell Growth

**Figure 2:**
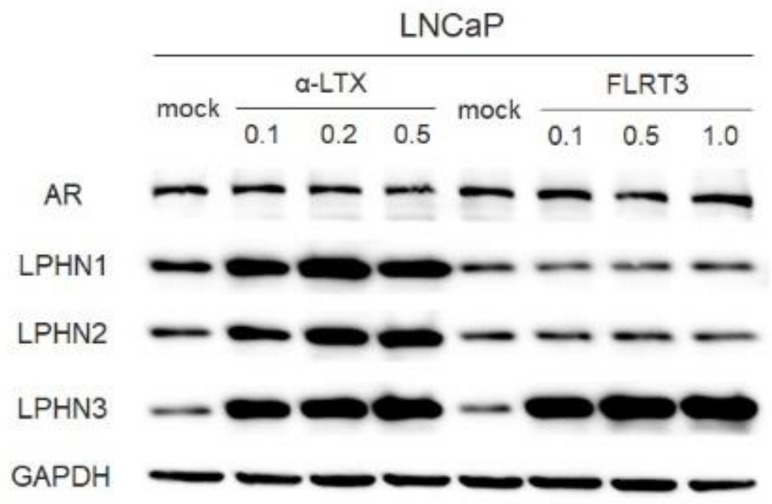
Effects of LPHN ligands on the expression of LPHNs in prostate cancer cells. Western blotting of AR (full-length), LPHN1, LPHN2, and LPHN3 in LNCaP cultured for 48 h with ethanol (mock), α-LTX (0.1–0.5 nM), or FLRT3 (0.1–1.0 nM), as indicated. GAPDH served as a loading control.

We next assessed the effects of LPHN ligands on the expression of LPHNs, as well as AR, in prostate cancer cells. Western blotting in LNCaP cells showed that α-LTX induced the expression of all three LPHNs and that FLRT3 induced that of only LPHN3 (Figure 2). α-LTX and FLRT3 did not considerably change the expression of full-length AR in LNCaP. Similarly, in C4-2 and PC-3, α-LTX increased the expression levels of LPHN1, LPHN2, and LPHN3, whereas FLRT3 increased those of LPHN3 only (Supplementary Figure S2).

**Figure 3:**
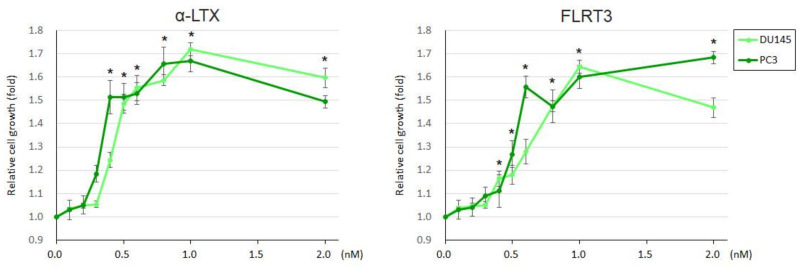
Effects of LPHN ligand treatment on the proliferation of prostate cancer cells. MTT assay in DU145 and PC-3 cultured for 96 h with ethanol (mock), α-LTX (0.1–2.0 nM), or FLRT3 (0.1–2.0 nM), as indicated. Cell viability representing the mean (±SD) from a total of 6 determinants is presented relative to that of the mock treatment in each line. * *p* < 0.05 (vs. mock treatment in both lines).

**Figure 4:**
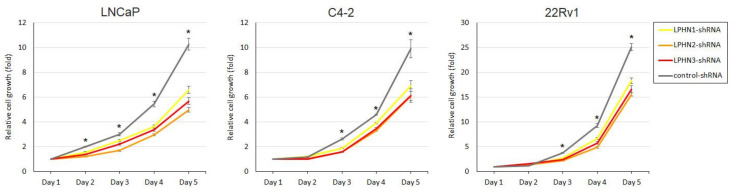
Effects of LPHN knockdown on the proliferation of prostate cancer cells. MTT assay in LNCaP, C4-2, and 22Rv1 sublines stably expressing control-shRNA, LPHN1-shRNA, LPHN2-shRNA, or LPHN3-shRNA and cultured for 24–96 h. Cell viability representing the mean (±SD) from a total of 6 determinants is presented relative to that of each subline at day 1. * *p* < 0.05 (vs. control-shRNA in all 3 sublines).

We then assessed the effects of LPHN ligands on the cell proliferation. In MTT assay, α-LTX (0.4–2.0 nM in all lines) and FLRT3 (0.5–1.0 nM in all lines) significantly induced the cell viability of DU145 and PC-3 (Figure 3), as well as LNCaP, C4-2, and 22Rv1 (Supplementary Figure S3), and the induction rates were slightly higher in AR-negative lines (highest in each agent: 67–72% by α-LTX; 65–68% by FLRT3) than in AR-positive lines (29–41% by α-LTX; 29–43% by FLRT3). By contrast, the knockdown of each LPHN resulted in significant decreases in the cell viability of LNCaP, C4-2, and 22Rv1 (Figure 4), as well as DU145 and PC-3 (Supplementary Figure S4), and the reduction rates (at day 5) were slightly higher in AR-positive lines (27–36% for LPHN1; 38–52% for LPHN2; 34–45% for LPHN3) than in AR-negative lines (15–20% for LPHN1; 33% for LPHN2; 23–26% for LPHN3). We also compared the effects of LPHN ligands on the proliferation of LPHN knockdown sublines (Supplementary Figure S5). The increased rates of cell viability by α-LTX were lower in LNCaP/PC-3 with LPHN1-shRNA (22%/37%), LPHN2-shRNA (22%/26%), or LPHN3-shRNA (32%/35%) than in LNCaP/PC-3-control-shRNA (57%/73%). However, the induction by FLRT3 was modest only in LPHN3 knockdown cells, while the increased rates were similar between control-shRNA and LPHN1-shRNA or LPHN2-shRNA sublines. Meanwhile, we compared the effects of androgen treatment on the proliferation of control vs. LPHN knockdown cells (Supplementary Figure S6). Although the effects of DHT on the viability of LNCaP-derived cells were still significant over the mock treatment in all the sublines examined, the induction rates were considerably higher in the control cells (2.54-fold) than in LPHN1 (1.42-fold), LPHN2 (1.36-fold), or LPHN3 (1.36-fold) knockdown cells.

**Figure 5:**
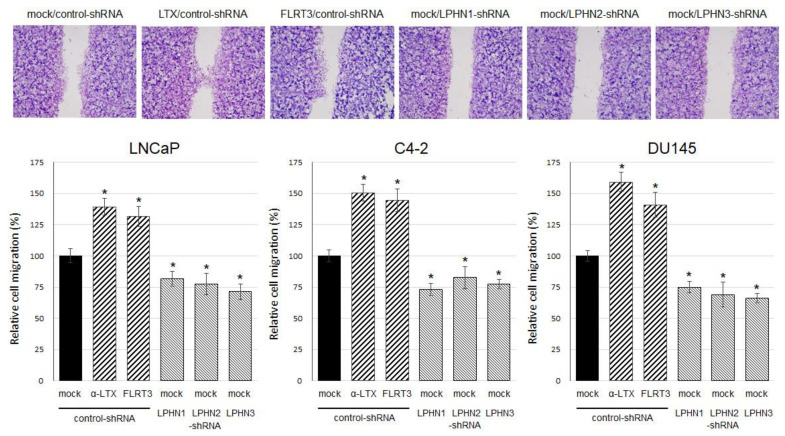
Effects of LPHN ligand treatment or knockdown on the migration of prostate cancer cells. Wound-healing assay in LNCaP, C4-2, or DU145 stably expressing control-shRNA, LPHN1-shRNA, LPHN2-shRNA, or LPHN3-shRNA and cultured for 24 h with ethanol (mock), 0.5 nM α-LTX, or 0.5 nM FLRT3, as indicated, after scratching. Cell migration determined by the rate of cells filling the wound area is presented relative to that of the control-shRNA subline with mock treatment. Each value represents the mean (+SD) from a total of 20 determinants. The scale bars under the images indicate 200 μm. * *p* < 0.05 (vs. mock treatment in control-shRNA subline).

We additionally assessed the impact of ligand treatment and knockdown on cell migration, using a scratch wound-healing assay. As expected, α-LTX/FLRT3 treatment and LPHN1/LPHN2/LPHN3 knockdown resulted in significant increases and decreases, respectively, in the cell migration of three lines (Figure 5).

### 2.3. Involvement of LPHNs in JAK-STAT Signaling

**Figure 6:**
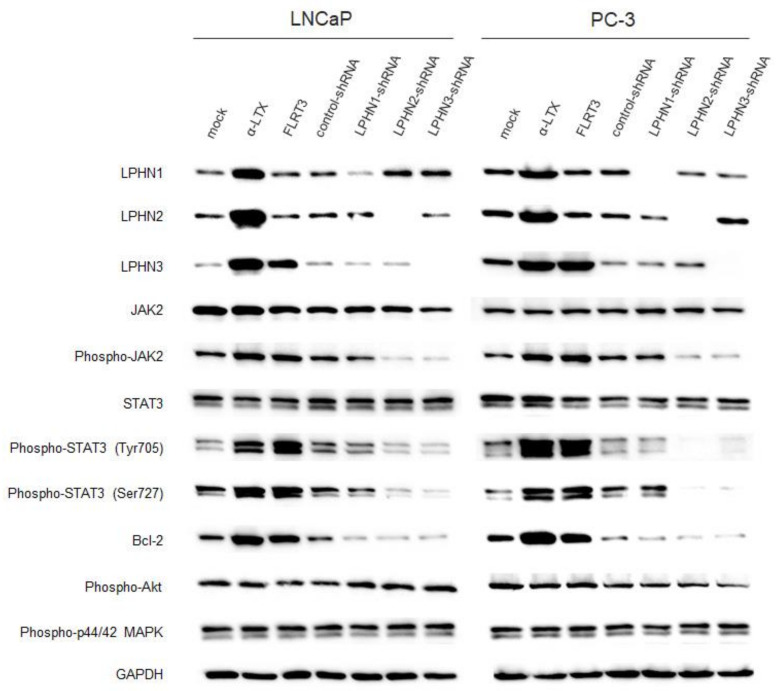
Effects of LPHN ligand treatment or knockdown on signal transduction pathways in prostate cancer cells. Western blotting of LPHN1, LPHN2, LPHN3, JAK2, phospho-JAK2, STAT3, phospho-STAT3, Bcl-2, phospho-Akt, and phospho-ERK1/2 in parental LNCaP or PC-3 line cultured for 24 h with ethanol (mock), 0.5 nM α-LTX, or 0.5 nM FLRT3, or in stable LNCaP or PC-3 subline expressing control-shRNA, LPHN1-shRNA, LPHN2-shRNA, or LPHN3-shRNA. GAPDH served as a loading control.

To explore the downstream signaling of LPHNs, we examined some of the signal transduction pathways known to involve prostate cancer progression and castration resistance [16], including JAK/STAT, Akt, and MAPK (Figure 6). In both LNCaP and PC-3 cells, α-LTX/FLRT3 treatment increased the expression levels of phospho-JAK2 and phospho-STAT3, as well as Bcl-2, whereas the knockdown of LPHN2 or LPHN3 resulted in their reduction. The effects of LPHN1 knockdown on the expression of phospho-JAK2 and phospho-STAT3 were modest. However, ligand treatment and LPHN knockdown did not considerably change the expression of phospho-Akt or phospho-ERK1/2.

### 2.4. Expression of LPHNs in Prostate Cancer Specimens

**Figure 7:**
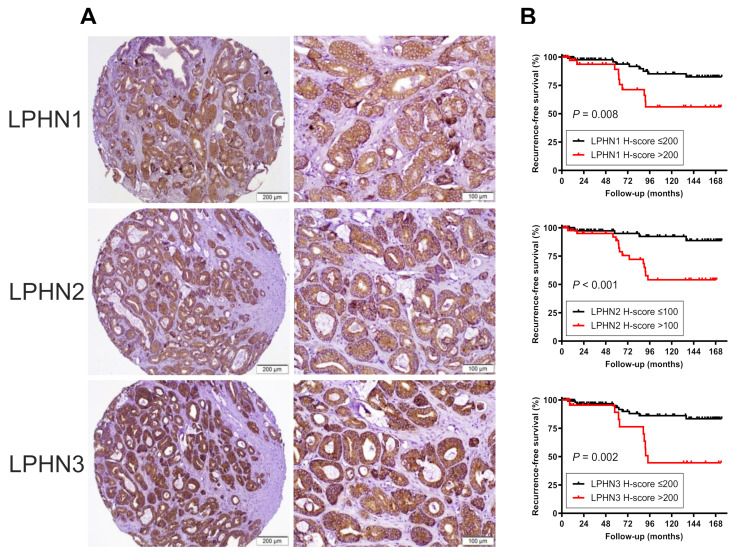
Immunohistochemistry of LPHNs in surgical specimens. (**A**) Representative images of LPHN1, LPHN2, and LPHN3 expression in prostatic adenocarcinoma. (**B**) Kaplan–Meier curves for recurrence-free survival in patients with LPHN1-low tumor (*n* = 113) vs. LPHN1-high tumor (*n* = 37), LPHN2-low tumor (*n* = 105) vs. LPHN2-high tumor (*n* = 45), or LPHN3-low tumor (*n* = 124) vs. LPHN3-high tumor (*n* = 24).

We stained immunohistochemically for LPHNs in sets of prostate tissue microarray (TMA) consisting of a total of 150 radical prostatectomy specimens. Positive signals were detected predominantly in the cytoplasm of non-neoplastic and neoplastic epithelial cells (Figure 7A).

The expression levels of LPHN1, LPHN2, and LPHN3 were significantly higher in prostate cancers than in corresponding non-neoplastic normal-appearing prostate tissues (Supplementary Table S1). When the levels of LPHN1 (200), LPHN2 (100), and LPHN3 (200) were dichotomized at their median H-scores, there were no significant differences in the clinicopathologic features of the patients, such as preoperative prostate-specific antigen (PSA), tumor grade, pT or pN staging category, and surgical margin status, between the two groups, except in significantly older age associated with high LPHN2 (Supplementary Table S2).

We then performed Kaplan–Meier analysis coupled with the log-rank test to assess possible associations of LPHN expression with postoperative oncologic outcomes. Patients with high LPHN1 tumor (*p* = 0.008), high LPHN2 tumor (*p* < 0.001), or high LPHN3 tumor (*p* = 0.002) had a significantly higher risk of biochemical recurrence (Figure 7B). To further determine if the expression levels of LPHNs were independent predictors of postoperative recurrence, multivariable analysis of histopathologic factors on radical prostatectomy, including LPHN immunohistochemistry data, was performed, using the Cox regression model. The elevated expression of LPHN1 [hazard ratio (HR) 3.118, 95% confidence interval (CI) 1.200–8.099, *p* = 0.020; Supplementary Table S3], LPHN2 (HR 7.022, 95% CI 2.247–21.94, *p* < 0.001; Supplementary Table S4), or LPHN3 (HR 3.370, 95% CI 1.255–9.050, *p* = 0.016; Supplementary Table S5) showed significantly worse recurrence-free survival.

## 3. Discussion

It is well known that G-protein–coupled receptors, as a large group of evolutionarily related proteins, mediate a variety of physiological and pathologic processes via crosstalk between signaling pathways [10,17,18]. By contrast, only limited data have suggested the involvement of LPHNs, a group of the G-protein–coupled receptors, in malignant tumors [10,19,20,21,22]. We herein investigated the functional role of LPHNs in the growth of prostate cancer, in relation to AR signaling, primarily via their activation (e.g., ligand treatment) and inactivation (e.g., knockdown) in cell line models.

We had originally identified LPHN3 as a potential AR target from our DNA microarray analysis in a control AR-positive bladder cancer UMUC3 cell line versus an AR-knockdown UMUC3 subline stably expressing AR-shRNA [23,24]. In UMUC3 cells, up-regulation of the expression of the *ADGRL3* gene and LPHN3 protein by androgen treatment was then confirmed [24]. In the present study, we demonstrated that androgen considerably induced the expression of not only LPHN3 but also LPHN1 and LPHN2 in AR-positive prostate cancer cells, which was at least partially blocked by an anti-androgen. ChIP assay in prostate cancer cells further revealed interactions of full-length ARs, as well as AR-V7 implicated in developing castration-resistant disease, with LPHN1, LPHN2, or LPHN3 at its promoter region, indicating the direct regulation of the expression of each LPHN by ARs. In addition, the effects of androgen on the viability of LNCaP-derived cells were diminished when LPHN1, LPHN2, or LPHN3 was knocked down. These findings, along with other data showing associations between the activity of LPHNs and cell growth, suggest that LPHNs represent downstream effectors of AR signaling in prostate cancer cells in both ligand-dependent and ligand-independent manners.

As mentioned earlier, LTX, a neurotoxin naturally found in the venom of widow spiders, is known to bind and activate the LPHNs [8,9,10]. In addition, FLRT3, a member of the fibronectin leucine-rich transmembrane protein family, has been documented to be an endogenous ligand for LPHN3 (and LPHN1) [10,12,25]. In prostate cancer cells, we demonstrated that α-LTX and FLRT3 induced the expression of all three LPHNs and only LPHN3, respectively. Treatment with α-LTX or FLRT3 was also found to induce the proliferation and migration of prostate cancer cells. Correspondingly, the knockdown of each LPHN resulted in a reduction in the cell proliferation (slightly fewer by LPHN1 than by LPHN2 or LPHN3) and migration (similar among 3 LPHNs). Meanwhile, it might be logical to see the more prominent effects of ligand treatment and knockdown on the cell growth in AR-negative (i.e., endogenously lower LPHN expression) and AR-positive (i.e., endogenously higher LPHN expression) lines, respectively.

The modulation of cancer development and progression, as well as chemotherapeutic resistance, by *ADGRLs*/LPHNs has been suggested. The observations in these studies include: (1) the elevated expression of LPHN2 and LPHN3 in breast cancer tissues, compared with normal breast tissues [21]; (2) elevated *ADGRL3* expression in acute myeloid leukemia cell lines possessing P-glycoprotein variants that are linked to chemoresistance [20]; (3) higher sensitivity to a chemotherapeutic agent cisplatin in gastric and colonic cancer cells with methylated *ADGRL2* [19]; and (4) an association of reduced *ADGRL3* expression in tumor tissues with shorter overall survival in patients with ependymoma [22]. In addition, loss-of-function mutations within *ADGRL* genes have been identified in several types of malignancy, including pulmonary, bladder, and ovarian cancers [10,26], suggesting their role as tumor suppressors. Signal transduction pathways downstream of LPHNs in cancer cells thus remain unrecognized. We here found that LPHNs induced the growth of prostate cancer cells and that the activation of LPHNs was associated with phosphorylation of JAK2/STAT3, but not Akt or ERK1/2, as well as the increased expression of Bcl-2. Supplementary Figure S7 illustrates the potential signaling pathways from AR-LPHNs to JAK2/STAT3 or Bcl-2 in prostate cancer cells to ultimately induce their growth and anti-apoptosis.

The expression status of *ADGRL* genes and LPHN proteins in prostate cancer remained unclear. In a study [21], the increased and decreased expression of LPHN2 and LPHN1/LPHN3, respectively, was shown in PC-3 cells, compared with their levels in THP-1 acute monocytic leukemia cells. We confirmed the expression of LPHNs in all the prostate cancer lines examined, and their levels were considerably higher in AR-positive lines, supporting the claim that LPHNs could represent downstream targets of AR signaling. Furthermore, we immunohistochemically investigated the expression of LPHNs in radical prostatectomy specimens and its prognostic significance. The expression of all three LPHNs was significantly elevated in prostate cancer, compared with adjacent normal-appearing prostate. Additionally, when their levels were dichotomized at respective median scores, there were no significant associations between the expression of LPHNs and the histopathology including the tumor grade and stage. Nonetheless, the elevated expression of LPHNs, as independent prognosticators, was associated with a significantly higher risk of postoperative recurrence. These findings in surgical specimens further support our data in cell lines indicating that LPHNs induce the progression of prostate cancer.

## 4. Materials and Methods

### 4.1. Cell Lines

Human prostatic carcinoma cell lines, LNCaP, C4-2, 22Rv1, DU145, and PC-3, were originally obtained from the American Type Culture Collection (ATCC) and then authenticated by the institutional core facility. The following ATCC-recommended cell culture media (all from Thermo Fisher Scientific, Waltham, MA, USA) were used: Roswell Park Memorial Institute 1640 medium for LNCaP and 22Rv1; Dulbecco’s modified Eagle’s medium for C4-2 and DU145; and Ham’s F-12K for PC-3. These parental cells were infected with control (sc-108080), LPHN1 (sc-45408-V), LPHN2 (sc-60919-V), or LPHN3 (sc-60921-V) shRNA lentiviral particles (all from Santa Cruz Biotechnology, Dallas, TX, USA), and the transduced cells were selected in culture medium containing 1–2 µg/mL puromycin (Sigma-Aldrich, St. Louis, MO, USA). All the parental lines and stable sublines were cultured in medium supplemented with 10% fetal bovine serum (FBS) and penicillin/streptomycin (both 100 units/mL) or in phenol red-free medium supplemented with 5% charcoal-stripped FBS for the experimental treatment with androgen/anti-androgen.

### 4.2. Chemicals and Antibodies

We purchased DHT from Sigma-Aldrich, ENZ from Selleckcem (Houston, TX, USA), α-LTX from Alomone Labs (Jerusalem, Israel), and recombinant human FLRT3 protein from R&D Systems (Minneapolis, MN, USA). Primary antibodies purchased were as follows: AR (clone 441, dilution for western blotting 1:1000; Santa Cruz Biotechnology); LPHN1 (clone A-4, 1:100; Santa Cruz Biotechnology); LPHN2 (clone E-3, 1:100; Santa Cruz Biotechnology); LPHN3 (clone B-6, 1:100; Santa Cruz Biotechnology); Bcl-2 (clone C-2, 1:500; Santa Cruz Biotechnology); JAK2 (clone D2E12, 1:1000; Cell Signaling Technology, Danvers, MA, USA)); phospho-JAK2 (Tyr1007/1008, 1:1000; Cell Signaling Technology); STAT3 (clone 124H6, 1:1000; Cell Signaling Technology); phospho-STAT3 (Tyr705 or Ser727, 1:1000; Cell Signaling Technology); phospho-Akt (Ser473, 1:2000; Cell Signaling Technology); phospho-p44/42 MAPK (ERK1/2) (Thr202/Tyr204, 1:2000; Cell Signaling Technology); GAPDH (clone 6C5, 1:5000; Santa Cruz Biotechnology); and AR-V7 (clone RM7; RevMAb Biosciences, Burlingame, CA, USA).

### 4.3. Western Blotting

Total proteins were extracted from the cells collected and washed twice with ice-cold 1× PBS with RIPA buffer supplemented with a protease and phosphatase inhibitor cocktail (Halt^TM^; Thermo Fisher Scientific), and the DC Protein Assay kit (Bio-Rad, Hercules, CA, USA) was used for the determination of protein concentration. Equal amounts of proteins (30 µg) obtained from the cell extracts were separated in 10% sodium dodecyl sulfate–polyacrylamide gel electrophoresis, transferred to polyvinylidene difluoride membrane electronically, blocked, and incubated with a specific antibody and a secondary antibody (anti-mouse or anti-rabbit IgG HRP-linked antibody; Cell Signaling Technology), followed by scanning with an imaging system (ChemiDoc™ MP, Bio-Rad).

### 4.4. ChIP Assay

We first performed a bioinformatic search [LASAGNA-Search 2.0 available online at https://biogrid-lasagna.engr.uconn.edu/lasagna_search/ (accessed on 11 November 2020) for potential AR binding sites in the promoters of *ADGRL1*, *ADGRL2*, and *ADGRL3*, and found target sites for each gene (see Figure 1B). A ChIP assay was then performed, using the Magna ChIP kit (Sigma-Aldrich) according to the manufacturer’s recommended protocol with minor modifications, as we described recently [27]. Briefly, cells were cross-linked with 1% formaldehyde for 10 min at room temperature, and the lysates were sonicated in nuclear buffer [four 30 s pulses, output 3.0, duty cycle 30% in ice with 120 s rest between pulses; Branson Sonifier 450 (Branson Ultrasonics, Danbury, CT, USA). Soluble chromatin was immunoprecipitated with an anti-AR antibody or normal mouse IgG (sc-2025; Santa Cruz Biochemistry) directly conjugated with protein A magnetic beads (Thermo Fisher Scientific). Immunoprecipitated DNA was eluted and reverse cross-linked, and DNA was extracted and purified using a spin filter column (Thermo Fisher Scientific). DNA samples were finally analyzed by PCR, using the following sets of primers: *ADGRL1* forward, TGCACAACCCTTCCAGATCT; *ADGRL1* reverse, TTTCCTTCCTTTCGCCTCCT; *ADGRL2* forward, TTGTGTACCTGGCCACTAATA; *ADGRL2* reverse, AATGAGGGACAGCGCAA; *ADGRL3* forward, AAAGAACCGAAGAGACAGCG; *ADGRL3* reverse, GAGCCACACAAACTCCTTCC. The PCR products electrophoresed on 1% agarose gel and stained with ethidium bromide were visualized using Gel Doc XR+ (Bio-Rad). 

### 4.5. MTT (3-(4,5-Dimethylthiazol-2-Yl)-2,5Diphenyltetrazolium Bromide) Assay

The MTT assay was used to evaluate the cell proliferation and drug response. Cells (1 × 10^3^) seeded in 96-well tissue-culture plates were cultured for up to 96 h in the absence or presence of α-LTX, FLRT3, or DHT and then incubated with 0.5 mg/mL of MTT (Sigma-Aldrich) in 100 μL medium for 4 h at 37 °C. MTT was dissolved by 150 μL of DMSO, and the absorbance was measured at a wavelength of 570 nm with background subtraction at 630 nm.

### 4.6. Wound-Healing Assay

The scratch wound-healing assay was adapted to evaluate the ability of cell migration. Cells at a density of ≥90% confluence in 6-well tissue-culture plates were scratched manually with a sterile 200 μL plastic pipette tip. The wounded monolayers of the cells were allowed to heal in serum-free medium for 24 h. The normalized cell-free area in photographed pictures (24 h/0 h) was then quantitated, using the ImageJ version 1.53 (National Institute of Health, Bethesda, MD, USA).

### 4.7. Prostate TMA and Immunohistochemistry

Two sets of TMA (75 cases in each set) consisting of retrieved prostate tissue specimens (i.e., adjacent normal-appearing prostate, prostatic adenocarcinoma) obtained by radical prostatectomy performed at the University of Rochester Medical Center in 2007 had been previously constructed upon appropriate approval from the Institutional Review Board [28,29]. None of the patients had received therapy with radiation, hormonal agents, and/or other anti-cancer drugs pre-operatively or post-operatively prior to clinical/biochemical recurrence defined as a single PSA level of ≥0.2 ng/mL or the introduction of adjuvant therapy.

Immunohistochemical staining was performed on the 5 µm sections, using a primary antibody to LPHN1 (dilution 1:100), LPHN2 (1:100), or LPHN3 (1:100), as we described previously [29,30]. All stains were manually assessed by 2 pathologists (Y.T. and H.M.) who were blinded to the sample identify. The H-score (0–300) [31] was calculated by multiplying the staining intensity (0/1/2/3) by the percentage of immunoreactive cells for each intensity.

### 4.8. Statistical Analysis

The Fisher exact test or chi-square test was used to determine the associations between categorized variables, whereas Student’s *t*-test was used to compare numerical data. Time-to-event estimates of recurrence-free survival were calculated by the Kaplan–Meier method and compared by the log-rank test. The Cox proportional hazards model was also used to evaluate the statistical significance of prognostic factors in a multivariable setting. All statistical analyses were performed, using EZR software version 1.55 [32] (R version 4.0.2; The R Foundation for Statistical Computing, Vienna, Austria) or Prism version 10.1.1 (GraphPad Software, Boston, MA, USA). A *p* value of less than 0.05 was considered to be statistically significant.

## 5. Conclusions

We identified LPHN1, LPHN2, and LPHN3 as downstream effectors of full-length ARs, as well as AR-V7, in prostate cancer cells. Moreover, LPHNs were found to promote the growth of not only androgen-sensitive prostate cancer but also castration-resistant or even AR-negative tumors, possibly via activating the JAK2/STAT3 pathway. Although no specific inhibitors are currently available, LPHNs may represent therapeutic targets for advanced prostate cancer.

## Data Availability

The data presented in this study are available on request from the corresponding author but are not publicly available due to privacy and/or ethical restrictions.

## Supplementary Materials

The following supporting information can be downloaded at: https://www.mdpi.com/article/10.3390/ijms25137289/s1.
