# Supplementary material for: Latrophilins as Downstream Effectors of Androgen Receptors including a Splice Variant, AR-V7, Induce Prostate Cancer Progression
Source: Int J Mol Sci. 2024 Jul 2;25(13):7289. doi: 10.3390/ijms25137289 (PMC11242678; doi:10.3390/ijms25137289)
Supplement: Supplementary file 1 [file ijms-25-07289-s001.zip › ijms-3001561-supplementary.pdf]

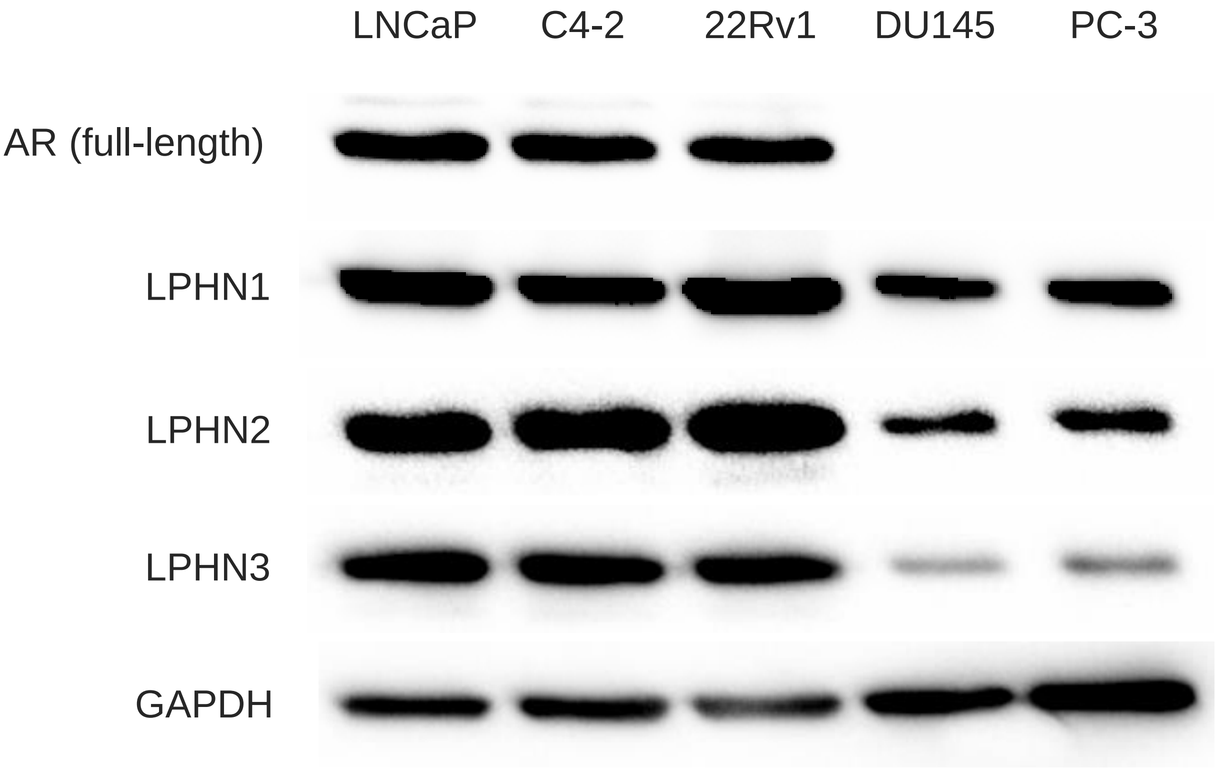

Supplementary Fig. S1. Expression of LPHNs in human prostate cancer cells. Western blotting of AR (full-length), LPHN1, LPHN2, and LPHN3 in parental cell lines. GAPDH served as a loading control.

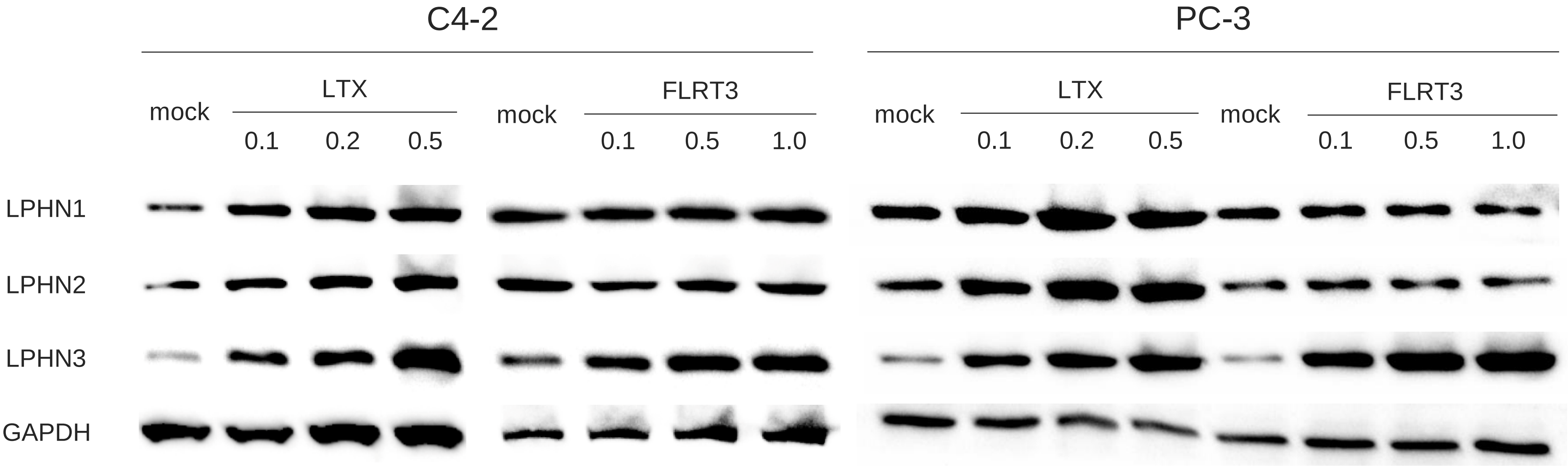

Supplementary Fig. S2. Effects of LPHN ligands on the expression of LPHNs in prostate cancer cells. Western blotting of LPHN1, LPHN2, and LPHN3 in C4-2 or PC-3 cultured for 48 hours with ethanol (mock), α-LTX (0.1-0.5 nM) or FLRT3 (0.1-1.0 nM), as indicated. GAPDH served as a loading control.

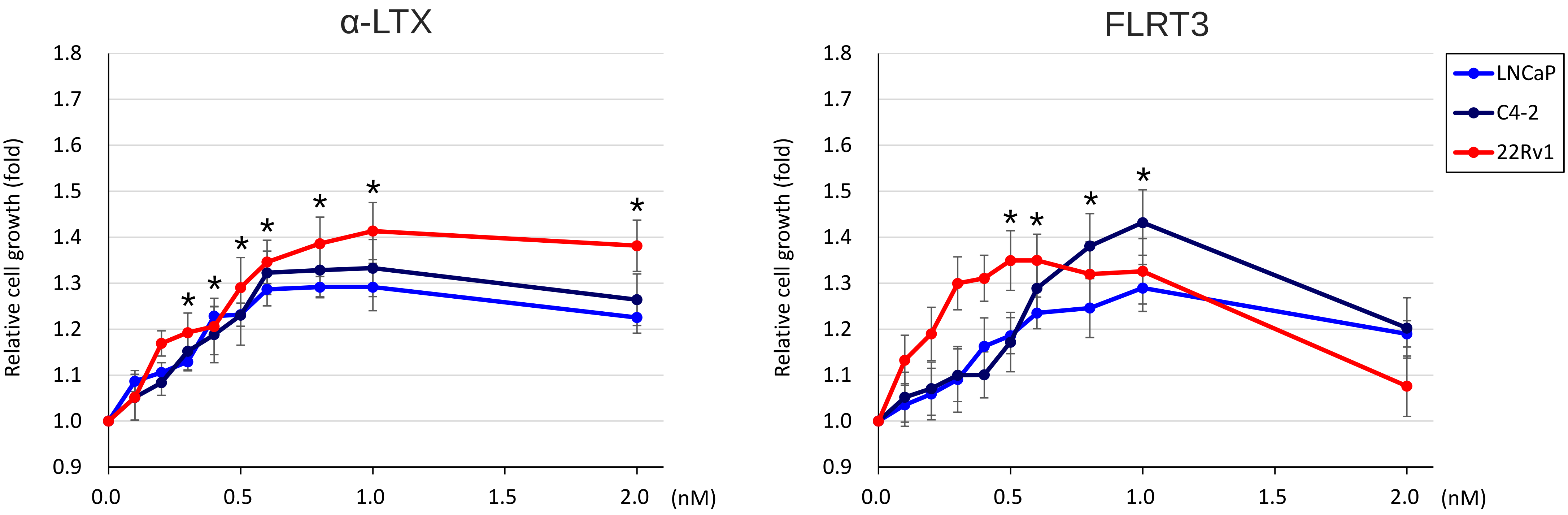

Supplementary Fig. S3. Effects of LPHN ligand treatment on the proliferation of prostate cancer cells. MTT assay in LNCaP, C4-2, and 22Rv1 cultured for 96 hours with ethanol (mock), α-LTX (0.1-2.0 nM), or FLRT3 (0.1-2.0 nM), as indicated. Cell viability representing the mean (± SD) from a total of 6 determinants is presented relative to that of mock treatment in each line. **P*<0.05 (vs. mock treatment in all 3 lines).

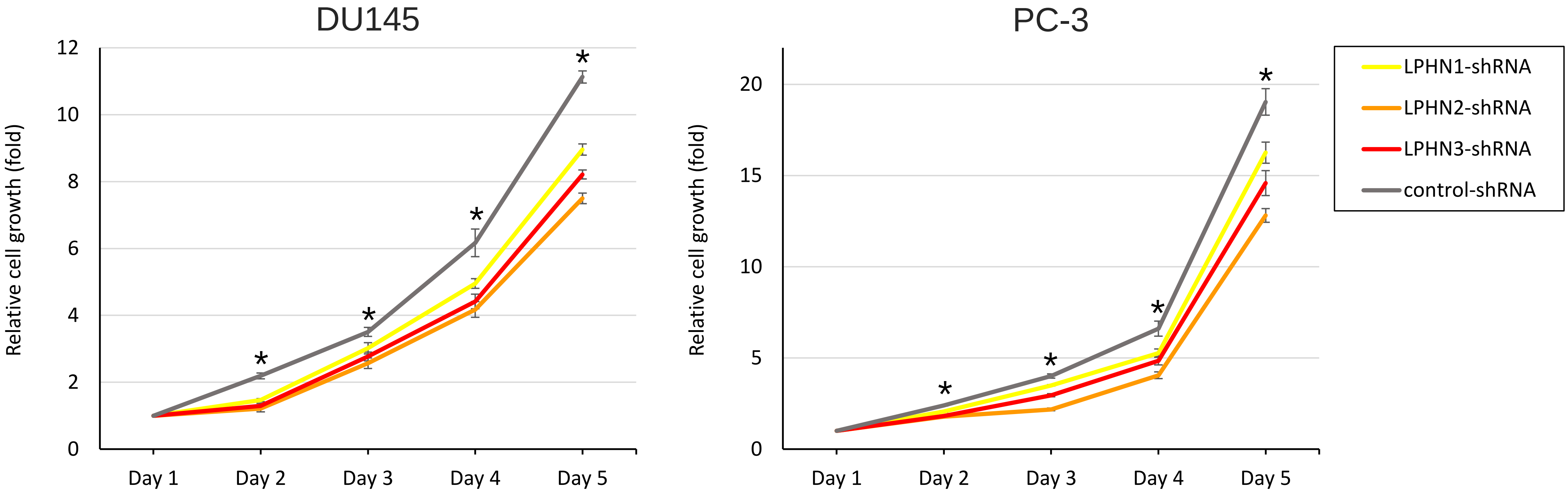

Supplementary Fig. S4. Effects of LPHN knockdown on the proliferation of prostate cancer cells. MTT assay in DU145 and PC-3 sublines stably expressing control-shRNA, LPHN1-shRNA, LPHN2-shRNA, or LPHN3-shRNA and cultured for 24-96 hours. Cell viability representing the mean (± SD) from a total of 6 determinants is presented relative to that of each line at day 1. **P*<0.05 (vs. control-shRNA in all 3 sublines).

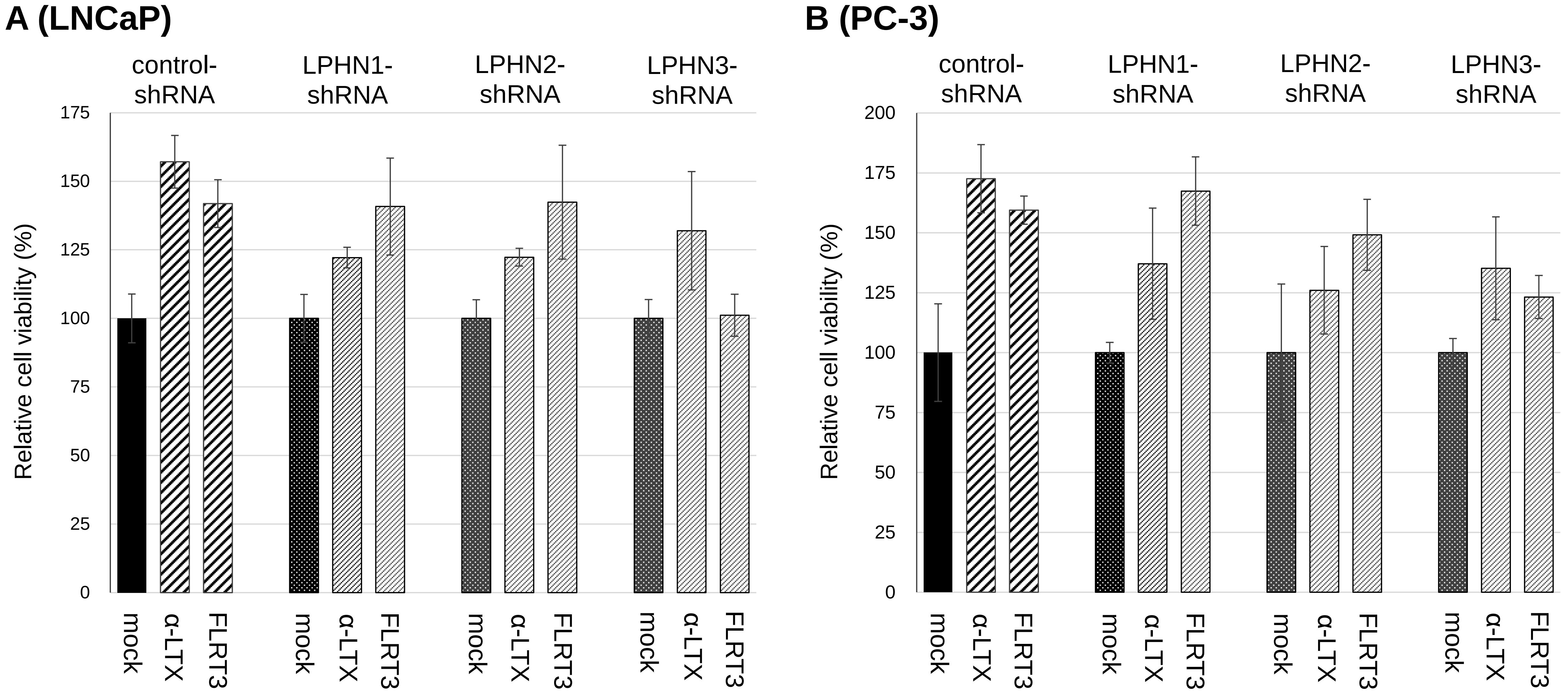

Supplementary Fig. S5. Effects of LPHN ligand treatment on the proliferation of LPHN knockdown prostate cancer cells. MTT assay in LNCaP (A) or PC-3 (B) sublines stably expressing control-shRNA, LPHN1-shRNA, LPHN2-shRNA, or LPHN3-shRNA and cultured for 96 hours with ethanol (mock), α-LTX (1 nM), or FLRT3 (1 nM), as indicated. Cell viability representing the mean (± SD) from a total of 6 determinants is presented relative to that of mock treatment in each subline.

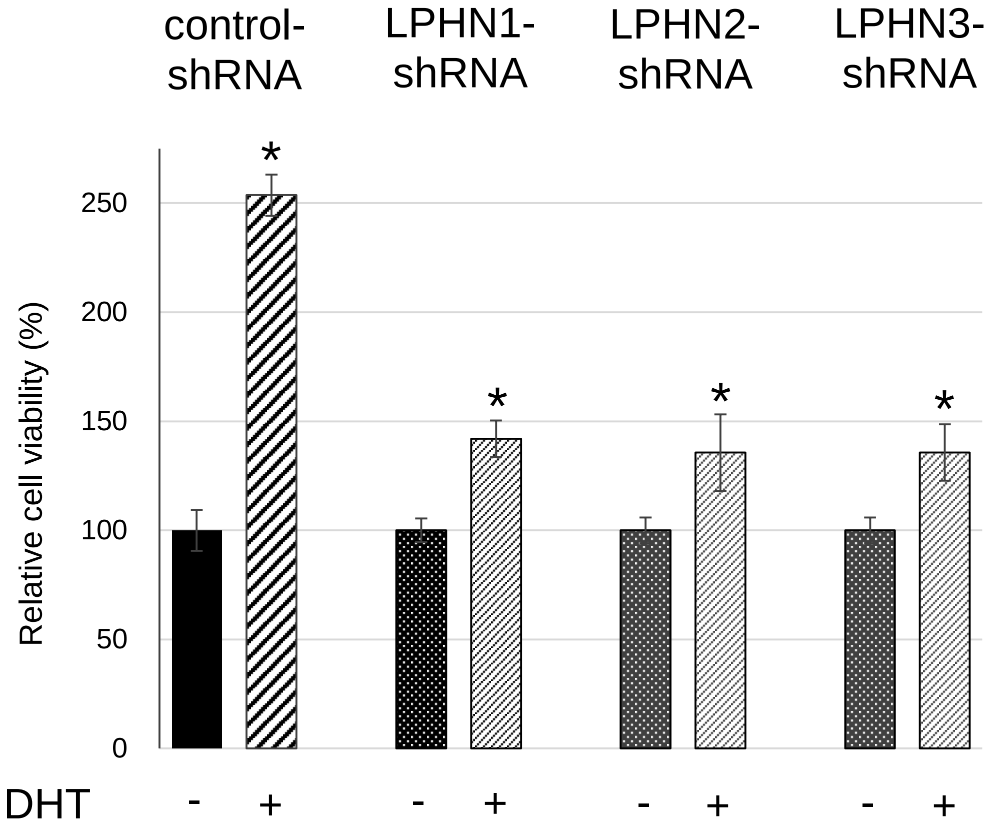

Supplementary Fig. S6. Effects of androgen treatment on the proliferation of LPHN knockdown prostate cancer cells. MTT assay in LNCaP sublines stably expressing control-shRNA, LPHN1-shRNA, LPHN2-shRNA, or LPHN3-shRNA and cultured for 48 hours with ethanol (mock) or DHT (10 nM), as indicated. Cell viability representing the mean (± SD) from a total of 6 determinants is presented relative to that of mock treatment in each subline. *P<0.05 (vs. mock treatment in each subline).

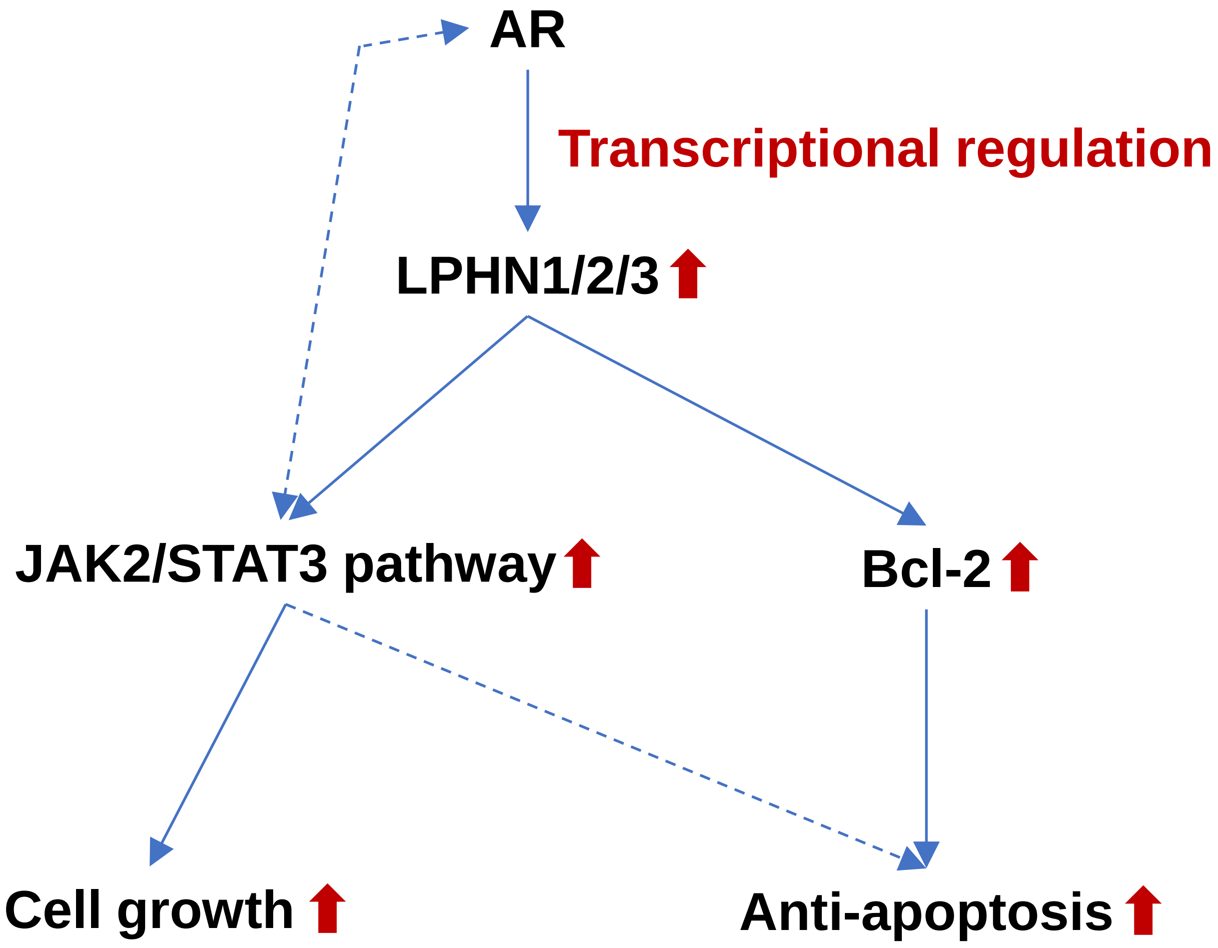

Supplementary Fig. S7. Potential signaling pathway involving prostate cancer progression. In prostate cancer cells, androgen receptor (AR) transcriptionally up-regulates the expression of latrophilins (LPHNs), including LPHN1, LPHN2, and LPHN3, leading to the activation of JAK2/STAT3 pathway, as well as Bcl-2.

Supplementary Table S1. H-scores of immunostaining for LPHNs in non-neoplastic prostate and prostatic adenocarcinoma.

	**Non-neoplastic**	**Carcinoma**	***P***
**LPHN1** (mean ± SD)	156 ± 52	217 ± 42	<0.001
**LPHN2** (mean ± SD)	86 ± 39	110 ± 63	<0.001
**LPHN3** (mean ± SD)	104 ± 51	194 ± 48	<0.001

Supplementary Table S2. Clinicopathologic features of patients according to the expression of LPHNs.

	**LPHN1** (H-score)		***P***	**LPHN2** (H-score)		***P***	**LPHN3** (H-score)[a]		***P***
	**≤200**	**>200**		**≤100**	**>100**		**≤200**	**>200**	
n	113	37		105	45		124	24	
**Age** (mean ± SD, year)	59.8 ± 6.9	61.2 ± 6.7	0.294	59.3 ± 7.1	62.0 ± 5.9	0.030	60.1 ± 4.9	60.7 ± 8.0	0.690
**Preoperative PSA** (mean ± SD, ng/mL)	6.00 ± 3.49	7.48 ± 6.99	0.091	6.01 ± 3.75	7.18 ± 6.18	0.156	6.55 ± 4.88	5.42 ± 3.05	0.276
**Grade Group**			0.634			0.447			0.239
1	28 (24.8%)	8 (21.6%)		28 (26.7%)	8 (17.8%)		32 (25.8%)	3 (12.5%)	
2	69 (55.8%)	19 (51.4%)		56 (53.3%)	26 (57.8%)		64 (51.6%)	17 (70.8%)	
3	11 (9.7%)	3 (8.1%)		10 (9.5%)	4 (8.9%)		13 (10.5%)	1 (4.2%)	
4	7 (6.2%)	5 (13.5%)		6 (5.7%)	6 (13.3%)		11 (8.9%)	1 (4.2%)	
5	4 (3.5%)	2 (5.4%)		5 (4.8%)	1 (2.2%)		4 (3.2%)	2 (8.3%)	
**pT**			0.470			0.310			0.243
2 / 2+	89 (78.8%)	26 (70.3%)		83 (79.0%)	32 (71.1%)		97 (78.2%)	16 (66.7%)	
3a	18 (15.9%)	8 (21.6%)		15 (14.3%)	11 (24.4%)		19 (15.3%)	7 (29.2%)	
3b	6 (5.3%)	3 (8.1%)		7 (6.7%)	2 (4.4%)		8 (6.5%)	1 (4.2%)	
**pN**			0.617[b]			0.340[b]			1.000[b]
0	69 (61.1%)	26 (70.3%)		63 (60.0%)	32 (71.1%)		81 (65.3%)	13 (54.2%)	
1	3 (2.7%)	2 (5.4%)		2 (1.9%)	3 (6.7%)		5 (4.0%)	0 (0%)	
X	41 (36.3%)	9 (24.3%)		40 (38.1%)	10 (22.2%)		38 (30.6%)	11 (45.8%)	
**Surgical margin**			0.544			0.566			0.725
Negative	102 (90.3%)	32 (86.5%)		95 (90.5%)	39 (86.7%)		111 (89.5%)	21 (87.5%)	
Positive	11 (9.7%)	5 (13.5%)		10 (9.5%)	6 (13.3%)		13 (10.5%)	3 (12.5%)	

PSA, prostate-specific antigen

[a] Cancer tissue is absent in 2 cases. [b] pN0 *vs.* pN1.

Supplementary Table S3. Multivariable analysis for LPHN1.

	HR	95% CI	*P*
**Grade Group**			
1 or 2	Reference		
3	2.255	0.632-8.049	0.210
4	4.448	1.160-17.05	0.030
5	5.659	1.294-24.74	0.021
**pT**			
2	Reference		
3a	5.150	1.535-17.28	0.008
3b	2.320	0.443-12.14	0.319
**Lymph node metastasis**	1.906	0.406-8.951	0.414
**Surgical margin**	1.195	0.317-4.498	0.792
**LPHN1** (H-score >200)	3.118	1.200-8.099	0.020

CI, confidence interval; HR, hazard ratio

Supplementary Table S4. Multivariable analysis for LPHN2.

	HR	95% CI	*P*
**Grade Group**			
1 or 2		Reference	
3	3.375	0.933-12.20	0.064
4	4.622	1.241-17.21	0.023
5	11.87	2.594-54.34	0.001
**pT**			
2		Reference	
3a	3.430	1.127-10.44	0.030
3b	3.061	0.487-19.25	0.233
**Lymph node metastasis**	2.331	0.420-12.93	0.333
**Surgical margin**	1.157	0.287-4.666	0.837
**LPHN2** (H-score >100)	7.022	2.247-21.94	<0.001

CI, confidence interval; HR, hazard ratio

Supplementary Table S5. Multivariable analysis for LPHN3.

	HR	95% CI	*P*
**Grade Group**			
1 or 2	Reference		
3	2.973	0.866-10.21	0.083
4	6.481	1.795-23.40	0.004
5	4.996	1.118-22.33	0.035
**pT**			
2	Reference		
3a	3.426	0.987-11.89	0.052
3b	2.397	0.405-14.19	0.335
**Lymph node metastasis**	1.054	0.201-5.525	0.951
**Surgical margin**	1.207	0.305-4.776	0.789
**LPHN3** (H-score >200)	3.370	1.255-9.050	0.016

CI, confidence interval; HR, hazard ratio
